# Cardiac proteomics reveals the potential mechanism of microtubule associated protein 4 phosphorylation-induced mitochondrial dysfunction

**DOI:** 10.1186/s41038-019-0146-3

**Published:** 2019-03-11

**Authors:** Lingfei Li, Junhui Zhang, Qiong Zhang, Yuesheng Huang, Jiongyu Hu

**Affiliations:** 1 Institute of Burn Research, State Key Laboratory of Trauma, Burns and Combined Injury, Southwest Hospital, Third Military Medical University (Army Medical University), Gaotanyan Street, Shapingba District, Chongqing, 400038 China; 2 Endocrinology Department, State Key Laboratory of Trauma, Burns and Combined Injury, Southwest Hospital, Third Military Medical University (Army Medical University), Gaotanyan Street, Shapingba District, Chongqing, 400038 China

**Keywords:** Microtubule associated protein 4, Mitochondria, Isobaric tag for relative and absolute quantitation

## Abstract

**Background:**

Our previous work suggested that microtubule associated protein 4 (MAP4) phosphorylation led to mitochondrial dysfunction in MAP4 phosphorylation mutant mice with cardiomyopathy, but the detailed mechanism was still unknown. Thus, the aim of this study was to investigate the potential mechanism involved in mitochondrial dysfunction responsible for cardiomyopathy.

**Methods:**

The present study was conducted to explore the potential mechanism underlying the mitochondrial dysfunction driven by MAP4 phosphorylation. Strain of mouse that mimicked constant MAP4 phosphorylation (S737 and S760) was generated. The isobaric tag for relative and absolute quantitation (iTRAQ) analysis was applied to the heart tissue. Gene Ontology (GO), Kyoto Encyclopedia of Genes and Genomes (KEGG), and protein-protein interaction (PPI) were all analyzed on the basis of differential expressed proteins (DEPs).

**Results:**

Among the 72 cardiac DEPs detected between the two genotypes of mice, 12 were upregulated and 60 were downregulated. GO analysis showed the biological process, molecular function, and cellular component of DEPs, and KEGG enrichment analysis linked DEPs to 96 different biochemical pathways. In addition, the PPI network was also extended on the basis of DEPs as the seed proteins. Three proteins, including mitochondrial ubiquitin ligase activator of NF-κB 1, reduced form of nicotinamide adenine dinucleotide (NADH)-ubiquinone oxidoreductase 75 kDa subunit, mitochondrial and growth arrest, and DNA-damage-inducible proteins-interacting protein 1, which play an important role in the regulation of mitochondrial function, may correlate with MAP4 phosphorylation-induced mitochondrial dysfunction. Western blot was used to validate the expression of the three proteins, which was consistent with iTRAQ experiments.

**Conclusions:**

These findings revealed that the DEPs caused by MAP4 phosphorylation in heart tissue using iTRAQ technique and may provide clues to uncover the potential mechanism of MAP4 phosphorylation-induced mitochondrial dysfunction.

**Electronic supplementary material:**

The online version of this article (10.1186/s41038-019-0146-3) contains supplementary material, which is available to authorized users.

## Background

Myocardial hypoxia/ischemia is a major risk factor for the development of chronic heart failure, an ultimate sequelae for many forms of heart diseases responsible for the leading cause of death worldwide [1, 2]. Accumulating data appear to support the argument that mitochondrial dysfunction contributes to the onset and progression of cardiac injury followed by heart failure under hypoxic and/or ischemic conditions [3]. Thus, a better understanding of the mechanism underlying cardiomyocyte mitochondrial dysfunction may therefore hopefully yield novel therapeutic targets against these cardiac disorders.

Microtubule associated protein 4 (MAP4) is recognized as a cytosolic protein that binds to tubulin and stimulates their polymerization [4, 5]. MAP4-microtubule binding is regulated through MAP4 phosphorylation, leading to the detachment of MAP4 from microtubules (MTs) and destabilization of the MT as a consequence [6]. We and others have reported that the phosphorylation sites of human MAP4 at S768 and S787 (corresponding to the S737 and S760 in mouse) are critical sites governing its detachment from MTs [7, 8]. Our previous study suggested that the phosphorylated MAP4 (p-MAP4) dissociated from MTs were translocated to mitochondria after hypoxia in cultured neonatal rat cardiomyocytes, which led to mitochondria dysfunction and apoptosis [7]. In addition, the aberrant MAP4 phosphorylation-induced cardiomyocyte mitochondrial dysfunction was still noted *in vivo* experiments and in heart tissues of patients of tetralogy of Fallot, and the MAP4 phosphorylation mutant mice developed cardiomyopathy at the age of 30–34 and 70–74 weeks, which mitochondrial dysfunction is deemed an important trigger for myocardial impairment and cardiac dysfunction [9]. However, the precise regulation that how MAP4 phosphorylation induced mitochondrial dysfunction is still elusive and it is worthwhile to explore the underlying mechanism.

Here, in this study, to investigate the differential expressed proteins (DEPs) of aberrant MAP4 phosphorylation *in vivo* and dig out the underlying mechanism that mediating MAP4 phosphorylation-induced mitochondrial dysfunction which is responsible for cardiomyopathy, we generated mice that mimicked MAP4 hyperphosphorylation at specific sites (S737 and S760). To avoid the complication and establish a potential cause-effect relationship, we studied relative young mice (24 weeks) with only initial injury, using an isobaric tag for relative and absolute quantitation (iTRAQ) followed by liquid chromatography-tandem mass spectrometric (LC-MS/MS) analysis. The significance of the findings with respect to deciphering the potential mechanism that involves MAP4 phosphorylation-induced mitochondrial dysfunction is discussed.

## Methods

### Mice

The mutant hyperphosphorylated MAP4 (S737 and S760) knock-in (MAP4 KI) mice were generated as previously described [9]. Animal experiments were performed in line with the United Kingdom Home Office and European Union guidelines and were authorized by the Animal Care Centre of the Army Medical University.

### Sample collection, protein extraction, and sodium dodecyl sulfate-polyacrylamide gel electrophoresis (SDS-PAGE) separation

Male wild type (WT) mice and male MAP4 KI littermates were sacrificed at 24 weeks of age (three mice per group), and the heart tissues were stored at − 80 °C. SDT buffer, including 1 mM dithiothreitol (DTT), 4% SDS, and 150 mM Tris-HCl, together with protease inhibitor, was added to the heart tissues, then the tissues were homogenized. After incubation in boiling hot water for 5 min, the mixture was sonicated at 4 °C and incubated for 15 min in 100 °C water. The homogenate was centrifuged at 14,000×*g* for 15 min at 4 °C, and the supernatant was collected and preserved at − 80 °C. The protein contents were examined with bicinchoninic acid (BCA) protein assay kit [10]. Subsequently, 20 μg samples with loading buffer were boiled for 5 min and separated on SDS-PAGE gel (12%, 250 V for 40 min). Coomassie blue staining was used to detect proteins.

### Protein processing and iTRAQ labeling

Protein processing was performed as described previously [11]. Briefly, 100 mM DTT was added to each sample and boiled in hot water for 5 min, then 200 μL UA buffer, including 150 mM Tris-HCl and 8 M urea, was added to the sample at room temperature, and the sample was centrifuged in a 30 kD ultrafiltration filter (Sartorius, Germany) for 15 min at 14,000×*g*, then 200 μL UA buffer was added and centrifuged again to abandon the filter solution. Subsequently, the filter was vibrated for 1 min at 600×*g* with 100 μL iodoacetamide (IAA) solution. After incubation for 30 min in the dark, the filter was centrifuged for 15 min at 14,000×*g* again and rinsed with 100 μL UA buffer for twice. Next, the filter was centrifuged with 100 μL dissolution buffer for twice using the previous condition. Finally, each filter that contained 4 μg trypsin and 40 μL dissolution buffer was vibrated for 1 min at 600×*g*, kept in 37 °C for 16–18 h, then the resultant peptides were obtained and moved to a new pipe and centrifuged for 15 min at 14,000×*g*. The peptide content was then analyzed at 280 nm.

Eight-plex iTRAQ reagent was used to label peptide mixture according to the instruction. For the iTRAQ experiments, two technical replicates and biological replicates (*n* = 3) were exerted per group.

### Peptide fractionation with reversed-phase chromatography

Agilent 1260 infinity II high performance liquid chromatography (HPLC) system was applied to remove interfering substances. Buffer A: 10 mM HCOONH_4_, 5% ACN, pH 10.0; buffer B: 85% ACN, 10 mM HCOONH_4_, pH 10.0. The peptides were eluted at a flow rate of 1 ml/min with a gradient of buffer B: 0% for 25 min, 0–7% for 5 min, 7–40% for 35 min, 40–100% for 5 min, and a final hold in 100% for 15 min. Elution was supervised by examining the absorbance at 214 nm, and the components were obtained per min (about 36 components). The samples were stored at − 80 °C [12, 13].

### LC-MS/MS analysis

Experiments were exerted on a Q Exactive mass spectrometer (Thermo Fisher, USA) that was coupled to Easy Nano Liquid Chromatography apparatus (nLC, Thermo Fisher, USA). The peptide mixture was transferred to a C18 reversed-phase column (nanoViper, Thermo Fisher, USA). The sample was subjected to the 60 min gradients of buffer B: 0–6% for 5 min, 6–28% for 40 min, 28–38% for 5 min, 38–100% for 5 min, and a final hold in 100% for 5 min. The data were collected using a data-dependent top 10 most abundant precursor ions from the survey scan (350–1800 m/z) for higher-energy collisional dissociation fragmentation. Survey scans were set to 70,000 resolution at 200 m/z, the target was 3e6 and the maximum fill time was 50 ms. The spectra resolution for higher-energy collisional dissociation was set to 17,500 at 200 m/z and the isolation window was 2 m/z. The normalized collision energy was 30 eV [12, 13].

### Data and bioinformatics analysis

MASCOT engine (version 2.5, Matrix Science, UK) in the Proteome Discoverer 2.1 (Thermo Electron, USA) was used for LC-MS/MS spectra analysis, screening against a mouse sequence database (Uniprot_MusMusculus_83374_20170809.fasta). The following parameters were applied; peptide mass tolerance was 20 ppm, fragment mass tolerance was 0.1 Da, trypsin with missed cleavages was 2, iTRAQ 8plex (N-term) with fixed modification, iTRAQ 8plex (K), variable modification of oxidation (M), and iTRAQ 8plex (Y). Ninety-nine percent confidence was applied for data analysis as determined by false discovery rate ≤ 1%.

The DEPs were defined as the following criteria: *p* < 0.05 and fold change ≤ 0.833 or ≥ 1.2. The DEPs were analyzed through the Gene Ontology (GO) platform (http://www.geneontology.org/), and protein grouping was analyzed based on functional notes using the GO terms for cellular component (CC), biological process (BP), and molecular function (MF) [14, 15]. The Kyoto Encyclopedia of Genes and Genomes (KEGG) platform (http://www.genome.jp/kegg/pathway.html) was used for pathway analysis [16, 17]. The Search Tool for the Retrieval of Interacting Genes/Proteins (STRING) software (http://string.embl.de/) was applied to protein-protein interaction (PPI) analysis. Cluster 3.0 software was applied to hierarchical cluster analysis.

### Validation by western blot (WB)

WB analysis was used to ensure the reality of the iTRAQ results. A tissue protein extraction reagent (T-PER, Thermo Scientific, USA) was applied to tissue homogenate. To discard insoluble protein, the mixture was centrifuged at 4 °C for 15 min by 16,000×*g*. SDS-PAGE gels (8–12%) were used for protein separation. After separation, proteins were moved to polyvinylidene fluoride (PVDF) membranes (Millipore, Germany) and followed by 5% nonfat-dried milk blocking, then incubated at 4 °C for 12–18 h with required antibodies. Target proteins were examined by chemiluminescence detection kit (GE Healthcare, USA). The antibodies were used as follows: mitochondrial ubiquitin ligase activator of NF-κB 1 (Mul1) (ab84067, Abcam), NADH-ubiquinone oxidoreductase 75 kDa subunit, mitochondrial (Ndufs1) (ab169540, Abcam), growth arrest and DNA-damage-inducible proteins-interacting protein 1 (Gadd45gip1) (16260-1-AP, Proteintech), MAP4 (Cat# A301-489A, Bethyl), and α-tubulin (11224-1-AP, Proteintech).

### Primary cell culture

Neonatal mouse cardiomyocytes and fibroblasts were cultured as our previous study described [9].

### Statistical analysis

The experimental data were showed as mean ± standard deviation (SD). The differential analysis of DEPs was tested by two-tailed Student’s *t* test, and *p* < 0.05 represented statistically significant.

## Results

### Protein identification and quantification

**Fig. 1:**
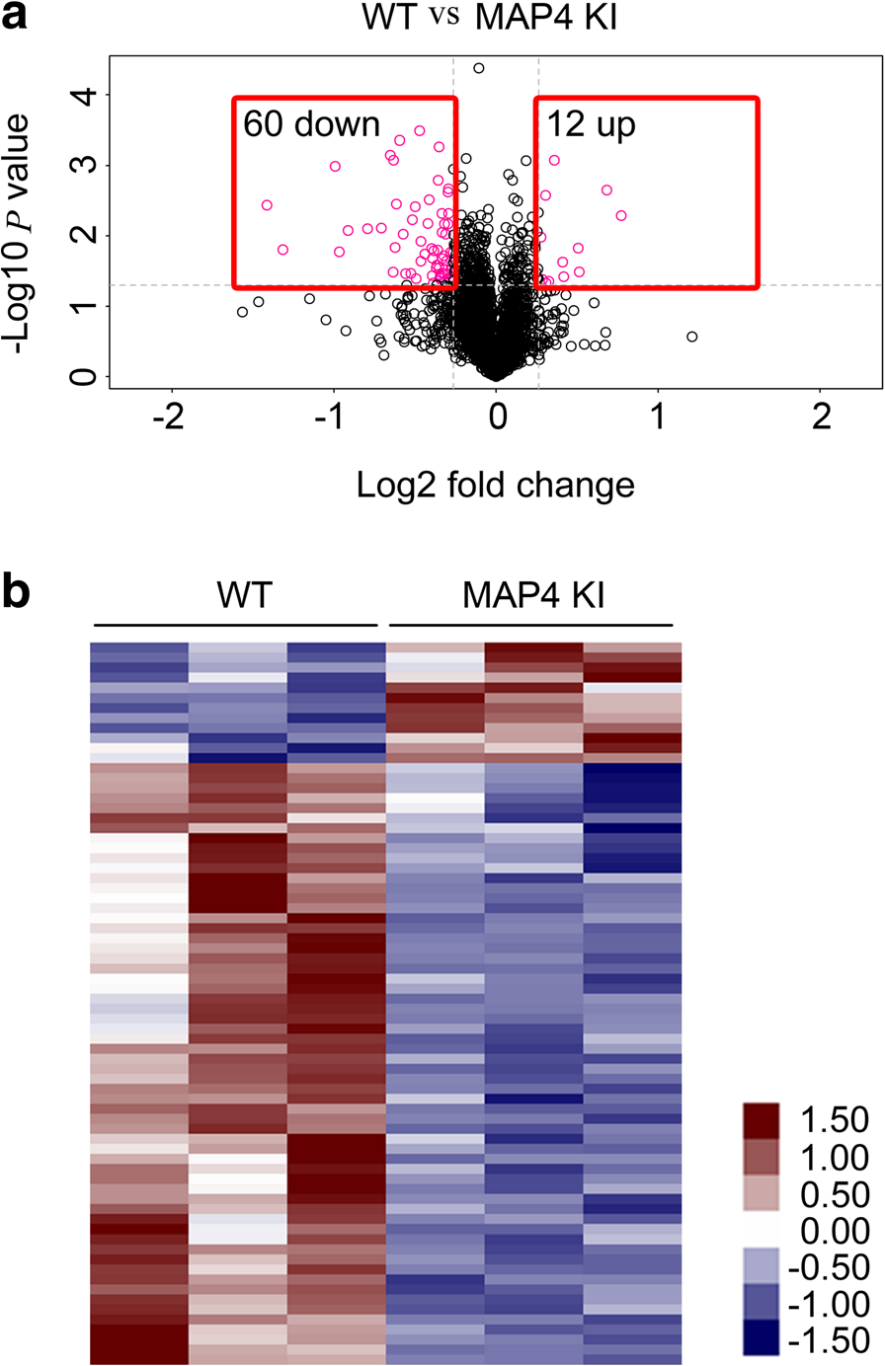
Protein identification and quantification between wild type (WT) and MAP4 (S737 and S760) knock-in (MAP4 KI) mice. **a** Volcano plot of cardiac differential expressed proteins (DEPs) in WT and MAP4 KI mice. The volcano plots were built using −log10 (*p* values) versus log2 (fold change). The upper left pink blots indicated 60 downregulated proteins and the upper right pink blots indicated 12 upregulated proteins. **b** Heat map of DEPs in WT and MAP4 KI mice. The color bar reveals the degree of relative changes in test samples of groups. Red indicated higher levels of protein expression, blue indicated lower levels of protein expression, and white indicated no significant changes in protein expression, as compared to WT mice. The darker the colors is, the greater the proteins change. The color scale exhibited at the lower right represented the fold change in protein expression

To elucidate the effect of MAP4 phosphorylation *in vivo*, a mouse strain that mimicked MAP4 hyperphosphorylation at specific sites (S737 and S760) was generated as described in our previous study [9]. A total of 23,520 unique peptides and 3812 proteins were identified. And 12 proteins were found to be upregulated and 60 were found to be downregulated in the cardiac tissue of MAP4 KI group compared with the control group (Additional file 1: Table S1). Volcano plots presented the probe sets in a graph of *p* values according to a given statistical test versus fold change. Typically, interesting features were located in the upper left (60 downregulated proteins) and right corners (12 upregulated proteins) of the graphs, as the fold change values (*x* axis) and *p* values (*y* axis) exceeded the usual thresholds used for analysis. In the present context, they represented the robust upregulated or downregulated cardiac proteins in the MAP4 KI mice compared with WT littermates (Fig. 1a). As shown in Fig. 1b, the hierarchical clustering analysis classified the proteins into two major clusters, which separated upregulated and downregulated proteins in each group. The resulting heat map also showed a clustering of the samples coming from two different groups.

### GO analysis of DEPs

**Fig. 2:**
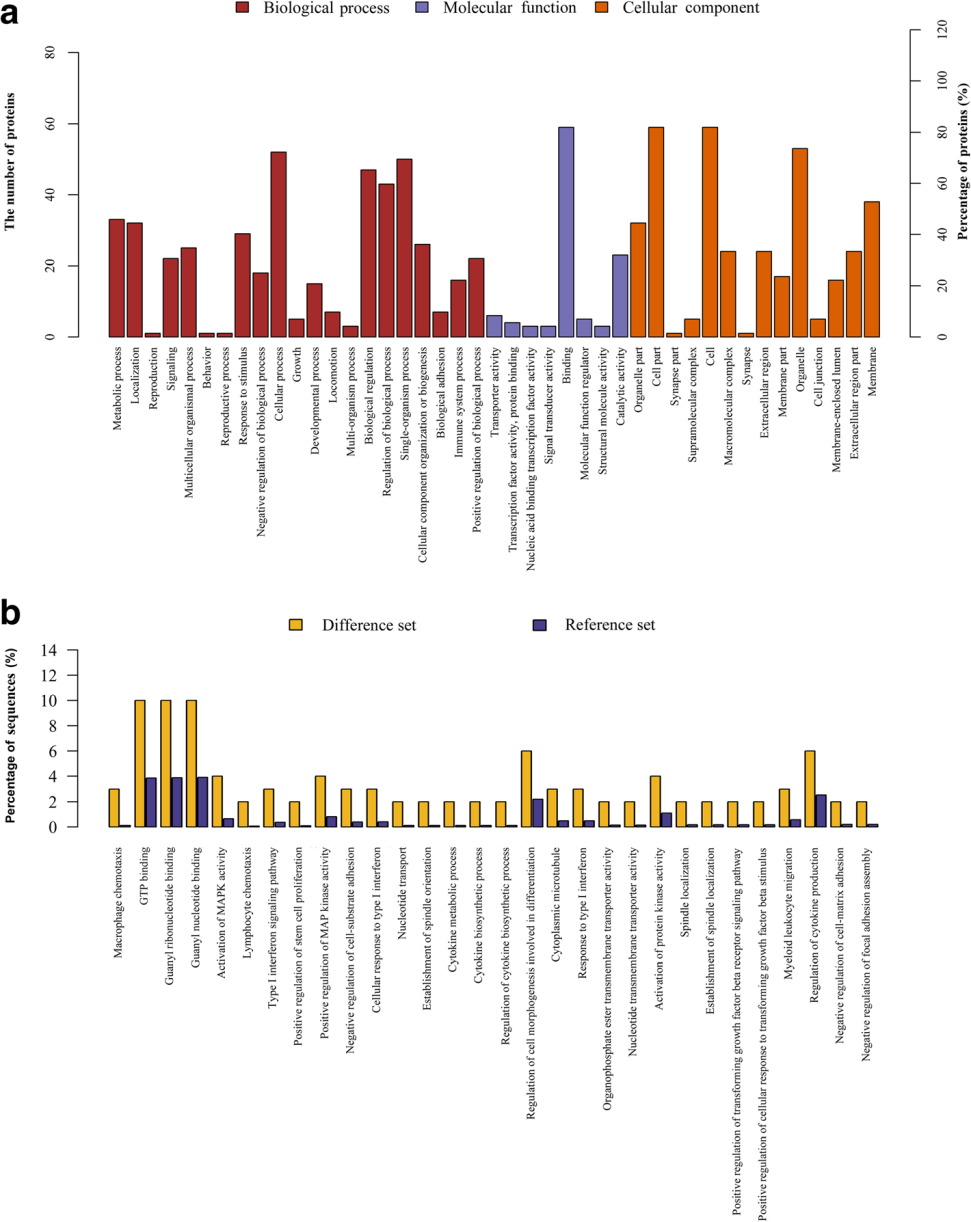
Gene Ontology (GO) analysis of differential expressed proteins (DEPs) between wild type (WT) and MAP4 (S737 and S760) knock-in (MAP4 KI) mice. **a** All identified proteins were classified according to the Biological process (BP), Molecular function (MF), and Cellular component (CC) based on the GO analysis. **b** GO enrichment analysis suggested that most DEPs identified were mainly enriched in the Guanosine triphosphate (GTP) binding, guanyl ribonucleotide binding, and guanyl nucleotide binding. All the data showed in this section have reached statistical significance (*p* < 0.05)

The 72 DEPs were classified using GO annotation. As shown in Fig. 2a, the main BPs of these proteins were cellular process, single-organism process, biological regulation process, and metabolic process. The main MFs were binding, catalytic activity, and transporter activity. The CCs of these proteins were mainly located in cell part, cell, membrane, and organelle part. Further analysis demonstrated that the most enriched GO terms including guanosine triphosphate (GTP) binding, guanyl ribonucleotide binding, and guanyl nucleotide binding (Fig. 2b).

### KEGG pathway analysis of DEPs

**Fig. 3:**
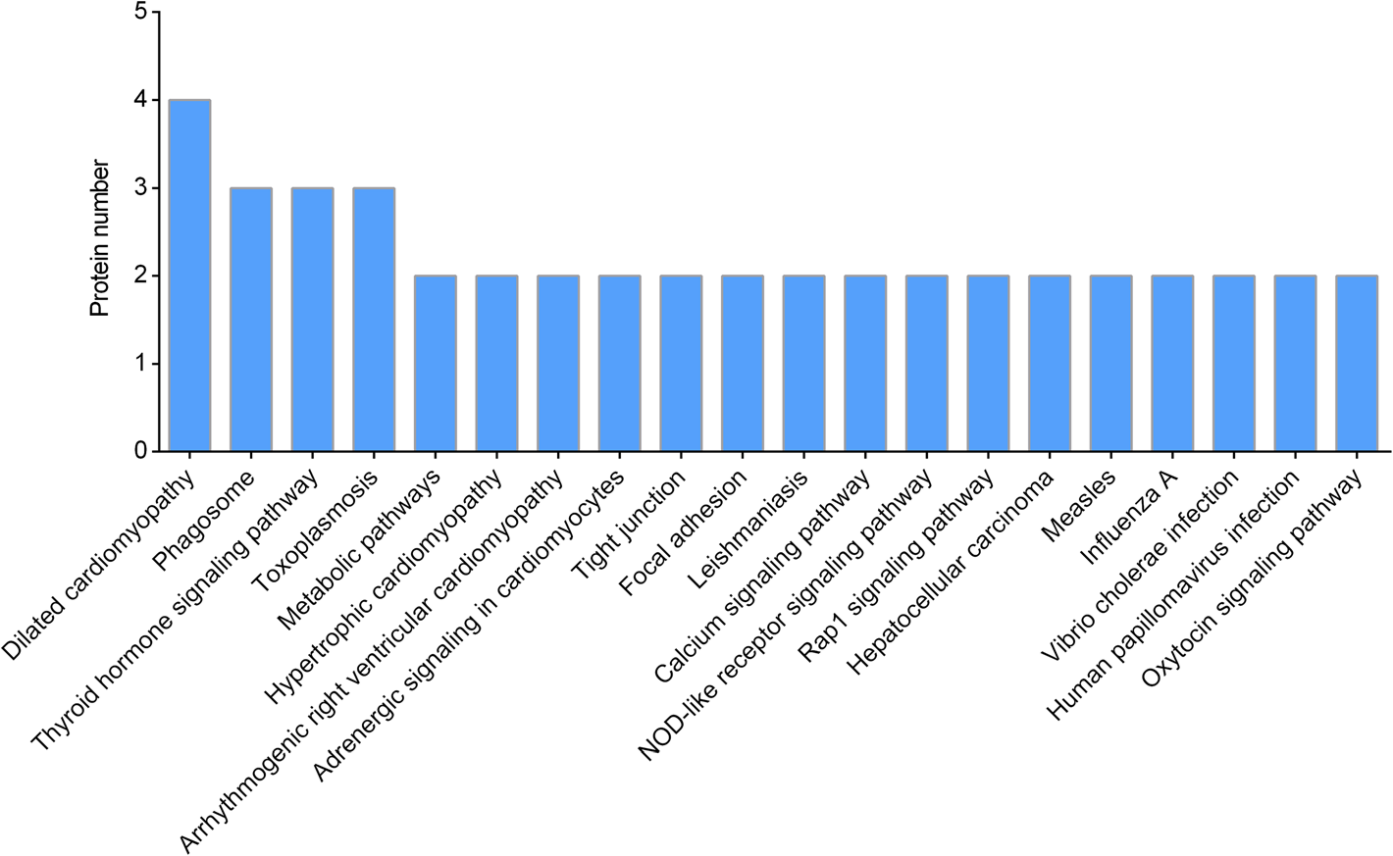
Kyoto Encyclopedia of Genes and Genomes (KEGG) pathway analysis of differential expressed proteins (DEPs) between wild type (WT) and MAP4 (S737 and S760) knock-in (MAP4 KI) mice. The top 20 pathways related to the DEPs were evaluated by KEGG analysis. Protein number involved the pathways was shown

The pathways related to DEPs were revealed on the basis of a KEGG pathway analysis. Ninety-six pathways were found to match (data not shown). Furthermore, KEGG pathway enrichment revealed that 20 pathways were enriched, and that these DEPs were mainly involved in dilated cardiomyopathy, phagosome, thyroid hormone signaling pathway, toxoplasmosis, metabolic pathways, hypertrophic cardiomyopathy, arrhythmogenic right ventricular cardiomyopathy, adrenergic signaling in cardiomyocytes, and so on (Fig. 3). It is notable that one of the most enriched pathways was the hypertrophic cardiomyopathy, which was identical to our previous work [9].

### PPI analysis

**Fig. 4:**
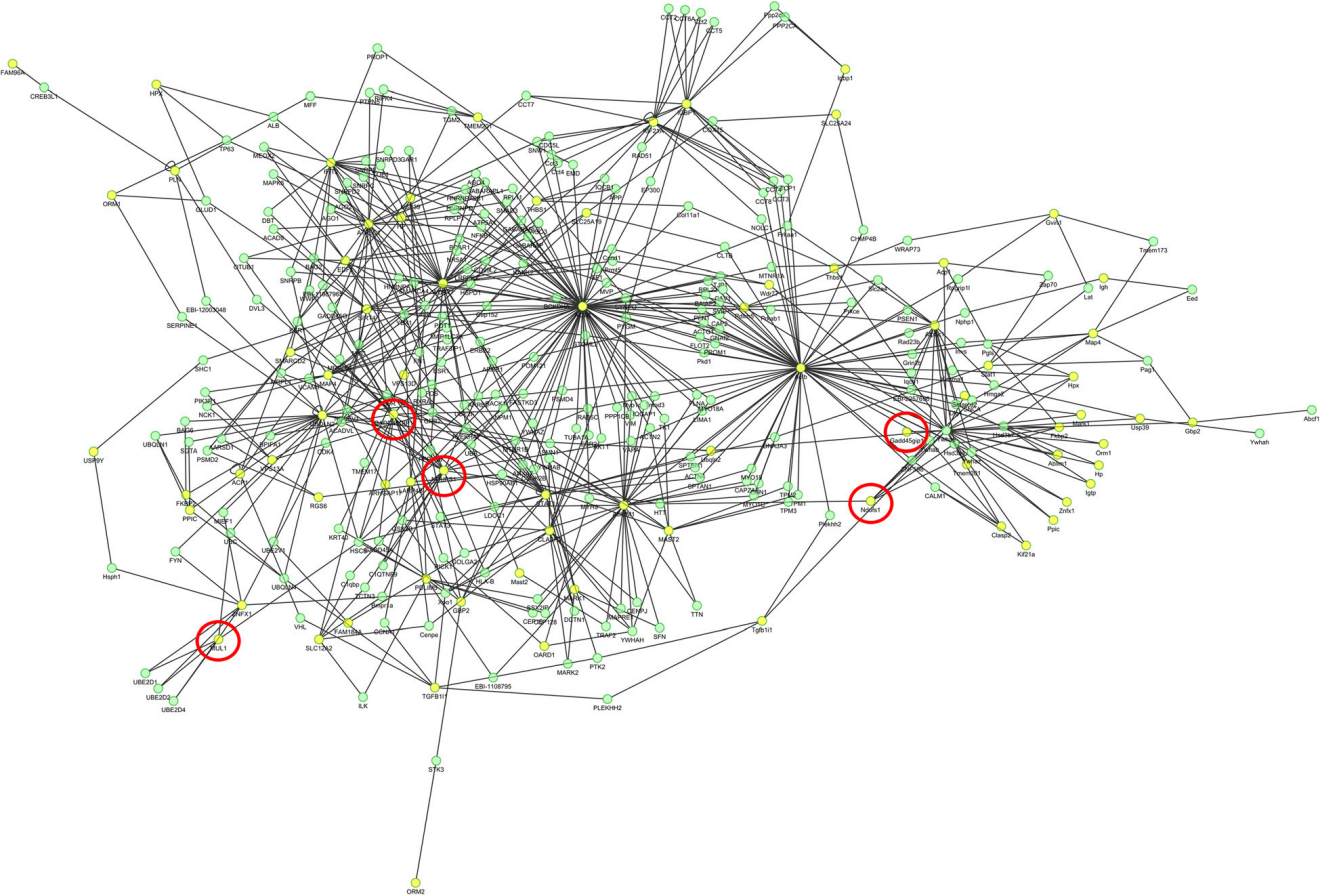
Protein-protein interaction (PPI) analysis. The interaction network of differential expressed proteins (DEPs) was analyzed using Search Tool for the Retrieval of Interacting Genes/Proteins (STRING) database. Yellow nodes represented target proteins and green nodes represented the related proteins which interact with the target proteins directly. Red circles indicated interesting proteins

The DEPs were regarded as seed proteins to construct an extended PPI network, which was not only consisted of the seed proteins, but also their direct PPI neighbors. We used STRING software to construct the network (Fig. 4), which revealed direct or indirect interactions between different proteins.

### WB validation of iTRAQ data

**Fig. 5:**
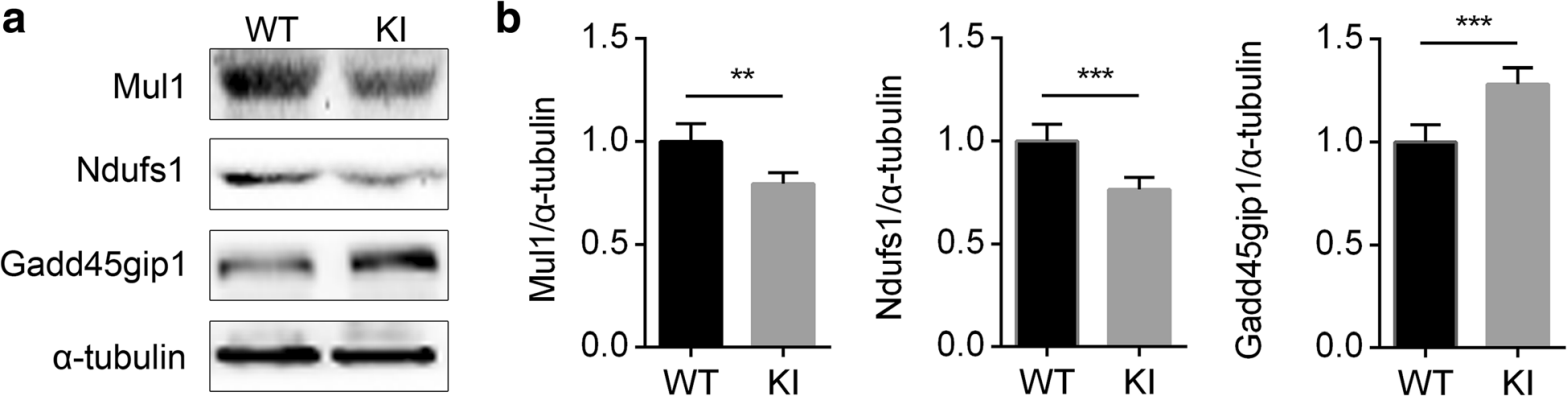
Western blot (WB) validation of isobaric tag for relative and absolute quantitation (iTRAQ) data between wild type (WT) and MAP4 (S737 and S760) knock-in (MAP4 KI) mice. **a**, **b** The protein expression of Mul1, Ndufs1, and Gadd45gip1 was validated between WT and MAP4 KI mice using WB analysis. KI, MAP4 KI. *n* = 6. Data were shown as mean ± standard deviation (SD). **p < 0.01 or ***p < 0.001 vs the WT group

To confirm the reliability of DEPs obtained by proteomics and further discern the mechanism of MAP4 phosphorylation-induced cardiomyocyte mitochondrial dysfunction, three DEPs, Mul1, Gadd45gip1, and Ndufs1, were chosen for WB, according to the aforementioned bioinformatics. The three proteins were involved in regulating mitochondrial function, and the results indicated that the Mul1 and Ndufs1 protein expression were significantly lower and Gadd45gip1 was higher in MAP4 KI mice compared with WT littermates, which was consistent with the iTRAQ results (Fig. 5 and Additional file 1: Table S1). In addition, to reveal that the differences of DEPs were truly between the hearts of these two genotypes of mice, we used primary cultured cardiomyocytes and fibroblasts, and demonstrated that MAP4 was mainly expressed in cardiomyocytes rather than fibroblasts (Additional file 1: Figure S1).

## Discussion

In our previous studies, we firstly demonstrated that p-MAP4 was a trigger factor that contributed to cardiomyocyte mitochondrial dysfunction, which led to the cardiac dysfunction and pathological remodeling [7, 9]. However, the detailed mechanism of how MAP4 phosphorylation triggered the mitochondrial dysfunction was still unclear. In the present study, for the first time, we investigated the DEPs between WT and MAP4 KI mice using iTRAQ-based global proteomics and LC-MS/MS analysis. We identified 72 DEPs in MAP4 KI mice.

In particular, several DEPs involved in mitochondrial dysfunction, which have not been reported correlated with MAP4, were identified and analyzed on the basis of PPI and their characteristics, thereby providing the underlying candidate molecular mechanisms that involved in MAP4 phosphorylation-induced mitochondrial dysfunction.

Further bioinformatics analysis, including GO analysis, KEGG assessment, and PPI were evaluated to reveal the characteristics of DEPs. The GO analysis revealed that cellular process, binding, and cell part were the most abundant categories in BP, MF, and CC, respectively. GTP binding, guanyl ribonucleotide binding, and guanyl nucleotide binding were the three most enriched GO terms. In KEGG analysis, several cardiomyopathy-related pathways, such as dilated cardiomyopathy, metabolic pathways, calcium signaling pathway, adrenergic signaling in cardiomyocytes, arrhythmogenic right ventricular cardiomyopathy, and hypertrophic cardiomyopathy were detected, indicating that MAP4 phosphorylation may correlate with these diseases. However, the detailed role of MAP4 phosphorylation in such diseases is still unknown.

Mitochondrial dysfunction [9, 18–20] has been recognized as one of the most important reasons for the development of cardiomyocyte injury. Our previous work revealed that MAP4 phosphorylation led to cardiomyocyte mitochondrial dysfunction under different stimuli (e.g., hypoxia, ischemia, and transverse aortic constriction) [7, 9, 21]. Here, in the results of KEGG analysis, both metabolic pathway and calcium signaling pathway were tightly correlated with the mitochondrial dysfunction, which may provide fundamental opening and evidence for future studies to reveal the mechanism of MAP4 phosphorylation-induced mitochondrial dysfunction. We also acquired some DEPs with thyroid hormone signaling pathway, adrenergic signaling in cardiomyocytes, arrhythmogenic right ventricular cardiomyopathy, nucleotide-binding oligomerization domain (NOD)-like receptor signaling pathway, and rap1 signaling pathway. We cannot exclude the possibility that these proteins may be triggers and have a certain capacity to switch on or exaggerate the role of key effector molecules, and further studies should be carried out.

In the current study, three mitochondrial proteins, including Mul1, Gadd45gip1, and Ndufs1, which were involved in the regulation of mitochondrial dysfunction, were chosen from DEPs to validate by WB. Mul1 has been known to be involved in the regulation of multiple proteins, including anti-apoptotic proteins, and is predominantly expressed in mitochondria of heart tissue [22]. The previous study reported that Mul1 interacted with multiple mitochondrial proteins, and overexpression of Mul1 promoted NF-κB activation, which increased the transcription of anti-apoptotic proteins and exhibited the inhibitory effects on mitochondrial apoptosis of cardiomyocyte, in contrast, the Mul1 inhibition led to susceptibility to mitochondrial apoptosis and dysfunction [23]. Ndufs1, the largest subunit of complex 1, which located at the mitochondrial inner membrane. The protein exhibited reduced form of nicotinamide adenine dinucleotide (NADH) dehydrogenase activity and oxidoreductase activity, which transferred electrons from NADH to the respiratory chain to exert oxidative phosphorylation [24]. The mutation or knockdown of Ndufs1 impaired mitochondrial oxygen consumption, increased reactive oxide species production, and decreased oxidative phosphorylation, contributing to progressive mitochondrial dysfunction followed by various diseases [25, 26]. Gadd45gip1 was newly identified as de novo components in large subunit of mitochondrial ribosome and played an important role in genomic stability, DNA repair, cell cycle regulation, and apoptosis. It was widely expressed in the heart, thyroid, and lymph node [27]. Previous data demonstrated that elevated Gadd45gip1 expression promoted activation of tumor protein p53 (p53), a pro-apoptotical protein, and its target genes in the suppression of cell growth and tumor development in human cancer cells [28]. The p53 was a downstream target of p38/mitogen-activated protein kinase (MAPK) and was known to induce mitochondrial dysfunction of cardiomyocyte upon the activation of p38/MAPK [29]. In our previous study, we have demonstrated that p38/MAPK activation induced MAP4 phosphorylation in cardiomyocyte [4]. Thus, we inferred that the MAP4 KI mice-induced increased Gadd45gip1 was also contaminated with p53 activation, which may be a pathway involved in cardiomyocyte mitochondrial dysfunction. Besides, in the DEPs, secretory protein including thrombospondin 1, and transcription factor, such as signal transducer and activator of transcription 1, have been reported to be associated with mitochondrial function [30, 31]. Whether they are associated with the observed changes of the above-mentioned three mitochondrial proteins are still unclear and deserve further research. The other secretory proteins, including immunoglobulin heavy variable 1–82, immunoglobulin heavy variable 1–26, transforming growth factor beta-1-induced transcript 1 protein, serum amyloid A-2 protein and serum amyloid A-1 protein, and transcription factors, such as NFX1-type zinc finger-containing protein 1 and endothelial differentiation-related factor 1, showed little correlation with cardiac mitochondrial function; while for other transcription factor, such as signal transducer and activator of transcription 1, has been reported to be associated with cardiac mitochondrial function [31]; however, the potential effect had little interaction with Mul1, Ndufs1, and Gadd45gip1, the detailed mechanism need to be investigated in future study.

In addition, the PPI analysis also revealed that 14-3-3 protein epsilon (Ywhae), which mediated signal transduction by binding to a multitude of functionally diverse signaling proteins, including phosphoserine-containing proteins, kinases, and transmembrane receptors [32, 33], was the center of the network in mice. In the network, Ywhae directly interacted with MAP4, Gadd45gip1, and Ndufs1, respectively. And Mul1 was interacted with Znfx1, which was also revealed a direct correlation with Ywhae. Furthermore, the protein expression of Mul1, Ndufs1, and Gadd45gip1 were validated by WB, which was consistent with the iTRAQ experiments. Thus, these findings provided a further link between MAP4 and Gadd45gip1, Ndufs1, and Mul1, suggesting possible molecular targets involved in MAP4 phosphorylation-induced cardiomyocyte mitochondrial dysfunction, and these screened DEPs may provide us new directions in mechanism exploration in future studies. Even though there are still some limitations in the present study, the screened proteins involved in mitochondrial function and other cardiac-specific function are limited to only a couple and the extent of change is less than twofold for most of these proteins, so the cause and effect relationship between these proteins and cardiac mitochondrial dysfunction still needs to be demonstrated in future studies. In addition, a relatively small number of DEPs were detected, which may be related to the young mice that we used, if we used elder mice, the phenotypes would be more obvious and the more DEPs may be detected.

## Conclusions

In conclusion, iTRAQ proteomic analysis was used to assess the cardiac protein expression profile, and 72 DEPs were detected in MAP4 KI mice, including 12 upregulated and 60 downregulated proteins as compared to WT littermates. GO analysis, KEGG assessment, and PPI analysis were all evaluated to reveal the characteristics of DEPs. And three proteins were chosen to validate, which were possibly correlated with MAP4 phosphorylation-induced cardiomyocyte mitochondrial dysfunction. Further study is warranted to clarify the precise mechanism for MAP4 phosphorylation-induced cardiomyocyte mitochondrial dysfunction.

## Additional file

Additional file 1: **Table S1.** The list of DEPs between WT and MAP4 KI by iTRAQ analysis. Figure S1. WB analysis of protein expression of MAP4 in primary mouse cardiomyocyte and fibroblast. *n* = 4. (DOCX 40 kb)

## Abbreviations

BP: Biological process
CC: Cellular component
DEPs: Differential expressed proteins
Gadd45gip1: Growth arrest and DNA-damage-inducible proteins-interacting protein 1
GO: Gene Ontology
GTP: Guanosine triphosphate
IAA: Iodoacetamide
iTRAQ: Isobaric tag for relative and absolute quantitation
KEGG: Kyoto Encyclopedia of Genes and Genomes
LC-MS/MS: Liquid chromatography-tandem mass spectrometric
MAP4: Microtubule associated protein 4
MAPK: Mitogen-activated protein kinase
MF: Molecular function
MT: Microtubule
Mul1: Mitochondrial ubiquitin ligase activator of NF-κB 1
NADH: Reduced form of nicotinamide adenine dinucleotide
Ndufs1: NADH-ubiquinone oxidoreductase 75 kDa subunit, mitochondrial
NOD: Nucleotide-binding oligomerization domain
p-MAP4: Phosphorylated MAP4
PPI: Protein-protein interaction
STRING: Search Tool for the Retrieval of Interacting Genes/Proteins
WB: Western blot
WT: Wild type
Ywhae: 14-3-3 protein epsilon
