# Supplementary material for: Cardiac proteomics reveals the potential mechanism of microtubule associated protein 4 phosphorylation-induced mitochondrial dysfunction
Source: Burns Trauma. 2019 Mar 11;7:8. doi: 10.1186/s41038-019-0146-3 (PMC6410511; doi:10.1186/s41038-019-0146-3)
Supplement: Supplementary file 1 — Table S1. The list of DEPs between WT and MAP4 KI by iTRAQ analysis. Figure S1. WB analysis of protein expression of MAP4 in primary mouse cardiomyocyte and fibroblast. n = 4. (DOCX 40 kb) [file 41038_2019_146_MOESM1_ESM.docx]

**Cardiac proteomics reveals the potential mechanism of microtubule associated protein 4 phosphorylation-induced mitochondrial dysfunction**

Lingfei Li, Junhui Zhang, Qiong Zhang, Yuesheng Huang^*^, Jiongyu Hu^*^

*To whom correspondence should be addressed. E-mail: [jiongyuhu@163.com](mailto:jiongyuhu@163.com) (Jiongyu Hu) or [yshuang1958@163.com](mailto:yshuang1958@163.com) (Yuesheng Huang).

**Additional file 1**

| **Table S1.** The list of DEPs between WT and MAP4 KI by iTRAQ analysis | | | |
| --- | --- | --- | --- |
| Accession | Gene Name | Description | Fold (KI/WT) |
| P01662 |  | Ig kappa chain V-III region ABPC 22/PC 9245 | 1.70758 |
| Q99LA6 | Igh | Igh protein | 1.60599 |
| Q3UD06 | Atp5c1 | ATP synthase subunit gamma | 1.42718 |
| Q8K3A6 | Armcx6 | Protein ARMCX6 | 1.41993 |
| A0A0B4J1J7 | Ighv1-82 | Immunoglobulin heavy variable 1-82 | 1.33554 |
| A0A087WPD1 | Rgs6 | Regulator of G-protein-signaling 6 | 1.32868 |
| A0A075B5V0 | Ighv1-26 | Immunoglobulin heavy variable 1-26 (Fragment) | 1.28224 |
| P30412 | Ppic | Peptidyl-prolyl cis-trans isomerase C | 1.25263 |
| A2AMD2 | Ankrd60 | Ankyrin repeat domain 60 | 1.23797 |
| Q9D8L4 |  | Uncharacterized protein | 1.23547 |
| Q8BMD8 | Slc25a24 | Calcium-binding mitochondrial carrier protein SCaMC-1 | 1.21976 |
| Q9CR59 | Gadd45gip1 | Growth arrest and DNA damage-inducible proteins-interacting protein 1 | 1.21157 |
| Q3UIA2 | Arhgap17 | Rho GTPase-activating protein 17 | 0.83237 |
| Q5H8C4 | Vps13a | Vacuolar protein sorting-associated protein 13A | 0.82927 |
| E9Q9C7 | Ablim1 | Actin-binding LIM protein 1 | 0.82507 |
| Q8R151 | Znfx1 | NFX1-type zinc finger-containing protein 1 | 0.82457 |
| B1ART2 | Vps13d | Vacuolar protein sorting 13D | 0.82346 |
| Q684Q9 |  | Ubiquitin-like protein GDX (Fragment) | 0.81738 |
| L7N451 | Gvin1 | Interferon-induced very large GTPase 1 | 0.81598 |
| Q03734 | Serpina3m | Serine protease inhibitor A3M | 0.81513 |
| Q80YQ1 | Thbs1 | Thrombospondin 1 | 0.81409 |
| Q9JMG1 | Edf1 | Endothelial differentiation-related factor 1 | 0.81403 |
| Q9CRC6 | Borcs7 | BLOC-1-related complex subunit 7 | 0.81378 |
| F8VPU6 | Usp9y | Ubiquitin-specific peptidase 9, Y chromosome | 0.80916 |
| P61014 | Pln | Cardiac phospholamban | 0.8056 |
| Q3U804 | Actb | Putative uncharacterized protein | 0.80204 |
| Q9Z0E6 | Gbp2 | Guanylate-binding protein 2 | 0.8018 |
| Q3TIX9 | Usp39 | U4/U6.U5 tri-snRNP-associated protein 2 | 0.80126 |
| E9PV66 | Myo18b | Myosin XVIIIb | 0.7994 |
| P45878 | Fkbp2 | Peptidyl-prolyl cis-trans isomerase FKBP2 | 0.79456 |
| A0A087WSU3 | Ndufs1 | NADH-ubiquinone oxidoreductase 75 kDa subunit, mitochondrial | 0.79402 |
| Q62219 | Tgfb1i1 | Transforming growth factor beta-1-induced transcript 1 protein | 0.79396 |
| B9EJA4 | Clasp2 | Clasp2 protein | 0.79319 |
| C9K101 | Mark1 | Non-specific serine/threonine protein kinase | 0.79158 |
| Q6A0A2 | Larp4b | La-related protein 4B | 0.78761 |
| E9Q1Q1 | Mast2 | Microtubule-associated serine/threonine-protein kinase 2 | 0.78518 |
| Q61249 | Igbp1 | Immunoglobulin-binding protein 1 | 0.78359 |
| Q8VCM5 | Mul1 | Mitochondrial ubiquitin ligase activator of NFKB 1 | 0.78306 |
| A0A0G2JFH2 | Map4 | Microtubule-associated protein (Fragment) | 0.78012 |
| Q99J09 | Wdr77 | Methylosome protein 50 | 0.77906 |
| E9Q0J5 | Kif21a | Kinesin-like protein KIF21A | 0.7762 |
| Q91X72 | Hpx | Hemopexin | 0.7741 |
| Q8R216 | Sirt4 | NAD-dependent protein lipoamidase sirtuin-4, mitochondrial | 0.76597 |
| Q9QZM0 | Ubqln2 | Ubiquilin-2 | 0.76441 |
| Q80XJ6 | Slc25a19 | Solute carrier family 25 (Mitochondrial thiamine pyrophosphate carrier), member 19 | 0.76167 |
| E9Q8P5 | Pdlim5 | PDZ and LIM domain protein 5 | 0.76056 |
| A0A0G2JFE9 | Ighv1-76 | Immunoglobulin heavy variable 1-76 (Fragment) | 0.75901 |
| A0A087WSP5 | Stat1 | Signal transducer and activator of transcription | 0.7508 |
| E9QM38 | Slc12a2 | Solute carrier family 12 member 2 | 0.74702 |
| A2A8U2 | Tmem201 | Transmembrane protein 201 | 0.7369 |
| Q60590 | Orm1 | Alpha-1-acid glycoprotein 1 | 0.72463 |
| Q9DCL2 | Fam96a | MIP18 family protein FAM96A | 0.72463 |
| Q64112 | Ifit2 | Interferon-induced protein with tetratricopeptide repeats 2 | 0.72096 |
| Q9Z1L5 | Cacna2d3 | Voltage-dependent calcium channel subunit alpha-2/delta-3 | 0.71017 |
| Q3UG43 | Oard1 | Putative uncharacterized protein | 0.70746 |
| Q3TAN6 | Irgm2 | Interferon inducible GTPase 2, isoform CRA_a | 0.69856 |
| A0A1W2P6K1 | Fam184a | Uncharacterized protein (Fragment) | 0.69396 |
| Q561M1 | Acp1 | Acp1 protein | 0.67851 |
| Q3UBS3 | Hp | Haptoglobin | 0.67196 |
| A0A0G2JDV3 | Gbp6 | Guanylate-binding protein 6 | 0.66159 |
| Q0GUM2 | Gm12250 | Interferon-gamma-inducible p47 GTPase | 0.65289 |
| Q9DCE9 | Igtp | Interferon gamma-induced GTPase | 0.64898 |
| P27546 | Map4 | Microtubule-associated protein 4 | 0.64474 |
| Q3TXH6 | Smarcd2 | Putative uncharacterized protein | 0.64321 |
| Q8R5A7 | BC023105 | cDNA sequence BC023105 | 0.6355 |
| U5NFV9 | Irga4 | Immunity-related GTPase family member a4 | 0.6116 |
| P07361 | Orm2 | Alpha-1-acid glycoprotein 2 | 0.57702 |
| U5NG72 | Irga6 | Immunity-related GTPase family member a6 | 0.53075 |
| P05367 | Saa2 | Serum amyloid A-2 protein | 0.51095 |
| U5NJE8 | Irga8 | Immunity-related GTPase family member a8 | 0.50238 |
| Q8VDW7 | Iigp1b | Interferon-inducible GTPase-like | 0.40154 |
| P05366 | Saa1 | Serum amyloid A-1 protein | 0.37489 |


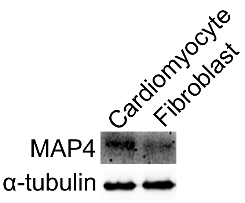


**Figure S1.** WB analysis of protein expression of MAP4 in primary mouse cardiomyocyte and fibroblast. n = 4.
